# Aerobic Granular Sludge–Membrane BioReactor (AGS–MBR) as a Novel Configuration for Wastewater Treatment and Fouling Mitigation: A Mini-Review

**DOI:** 10.3390/membranes11040261

**Published:** 2021-04-04

**Authors:** Riccardo Campo, Claudio Lubello, Tommaso Lotti, Gaetano Di Bella

**Affiliations:** 1 Department of Civil and Environmental Engineering—(DICEA), University of Florence, 50100 Florence, Italy; riccardo.campo@unifi.it (R.C.); claudio.lubello@unifi.it (C.L.); tommaso.lotti@unifi.it (T.L.); 2 Faculty of Engineering and Architecture, University of Enna “Kore”, 94100 Enna, Italy

**Keywords:** membrane bioreactor, aerobic granular sludge, EPS, fouling, AGS–MBR

## Abstract

This mini-review reports the effect of aerobic granular sludge (AGS) on performance and membrane-fouling in combined aerobic granular sludge–membrane bioreactor (AGS–MBR) systems. Membrane-fouling represents a major drawback hampering the wider application of membrane bioreactor (MBR) technology. Fouling can be mitigated by applying aerobic granular sludge technology, a novel kind of biofilm technology characterized by high settleability, strong microbial structure, high resilience to toxic/recalcitrant compounds of industrial wastewater, and the possibility to simultaneously remove organic matter and nutrients. Different schemes can be foreseen for the AGS–MBR process. However, an updated literature review reveals that in the AGS–MBR process, granule breakage represents a critical problem in all configurations, which often causes an increase of pore-blocking. Therefore, to date, the objective of research in this sector has been to develop a stable AGS–MBR through multiple operational strategies, including the cultivation of AGS directly in an AGS–MBR reactor, the occurrence of an anaerobic-feast/aerobic-famine regime in continuous-flow reactors, maintenance of average granule dimensions far from critical values, and proper management of AGS scouring, which has been recently recognized as a crucial factor in membrane-fouling mitigation.

## 1. Introduction and Global Overview

Presently, the most applied wastewater treatment technology in the world is represented by conventional activated sludge (CAS). This system often fails to remove nutrients to acceptable levels, and also produces large volumes of sludge that need further stabilization and whose disposal constitutes one of the highest operational costs [1].

Membrane bioreactors (MBRs) have emerged as the technology of choice for wastewater treatment of CAS [2,3]. MBRs combine a biological treatment and a physical solid–liquid separation using membrane filtration, and are becoming widely adopted for the treatment and reclamation of both municipal and industrial wastewater [2]. This is due to multiple advantages such as the generation of high-quality and largely disinfected effluent, capability to withstand high organic loading rates (OLRs), shorter hydraulic retention times (HRTs), capability to maintain longer solid retention time (SRT), resulting in less sludge production (low observed yield—“Yobs”), and high potentiality to biodegrade more recalcitrant substrates due to the prolonged acclimatization of microorganisms. Furthermore, the presence of membranes in the wastewater treatment chain eliminates the need for secondary clarifiers. This results in a significantly reduced footprint [4].

However, membrane-fouling remains the major drawback hampering the wider application of the MBR [3,5]. Fouling in MBRs refers to biofouling/organic and inorganic fouling [3,6]. Biofouling is commonly referred to as the attachment and growth of bacteria on the membrane surface, as well as the adsorption of bacterial byproducts, extracellular polymeric substances (EPS), and soluble microbial products (SMP) on the membrane surface (cake deposition) and inside membrane pores (pore-blocking), respectively. Biofouling is otherwise known as biocake or biofilm. Inorganic fouling, also known as scaling, is due to chemical precipitation of inorganic species and/or biological precipitation of inorganic–organic complexes [3]. Inorganic particulates in the mixed-liquor suspended solids (MLSS) also attach to the membrane surface or inside membrane pores to cause inorganic fouling [7]. Biofouling due to bacteria and their secreted EPSs has been identified as a major contributor to membrane-fouling in MBRs [3,8]. Fouling in MBRs is mitigated through different strategies that can significantly decrease operation (energy consumption) and maintenance costs [9]. Recently, great interest has been expressed in the introduction of granular biomass, such as that from aerobic granular sludge (AGS) technology, to mitigate membrane-fouling in MBRs systems [9,10,11].

AGS technology is a novel promising process consisting of a microbial aggregation of numerous self-immobilized functional microorganisms with diversified microbial communities and a tightly compact structure [12]. Compared to CAS, AGS shows several advantages, including higher settleability, stronger microbial structure, higher resilience to toxic/recalcitrant compounds of industrial wastewater [13], very good ability to handle high organic and shock-loading rates, great biomass concentration, large relative density, and the possibility to simultaneously degrade organic carbon and nutrients [14]. This technology can also substantially reduce sludge production [14] and land-space requirements. AGS systems were born of Sequencing Batch Reactor (SBR) processes, operating in full aerobic conditions, given the peculiarity of guaranteeing an alternation between abundance (feast) and starvation (famine) of the substrate that promotes the secretion of extracellular polymeric substances (EPSs) as the main substances that favor microbial aggregation in granular form [12,15]. EPSs secreted by microorganisms during the granulation step help to initiate the aerobic granulation process by bringing the bacterial cells and other particulate matter into an aggregate and playing a significant role in maintaining the structural integrity of AGS [16].

However, the main drawback of AGS technology is represented by the deterioration of granule stability under long-term operation, thus implying the breakage of granules with the consequent loss of solids in the effluent [17].

**Figure 1:**
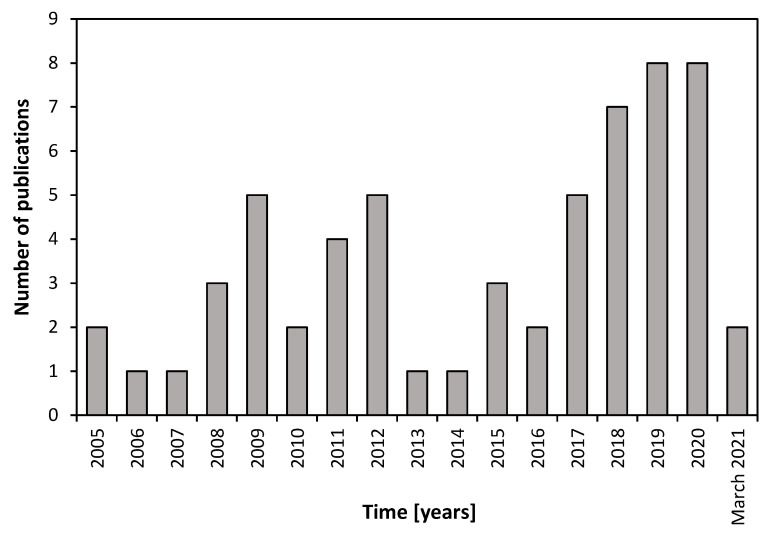
Survey of the number of published research papers since 2005 on membrane bioreactors (MBRs) and aerobic granular sludge (AGS) alone, and on AGS–MBR configuration. Data were acquired from the Scopus advanced research system by using the keywords “MBR”, “AGS”, and “AGS–MBR” (data were retrieved from Scopus^®^, Elsevier).

Over the years, numerous studies on the AGS process have contributed to perfecting the AGS process (see Figure 1), so much so that granular technology is currently applied at full scale in over 70 full-scale plants worldwide [12]. Among the essential operating conditions for the cultivation and maintenance of stable granular biomass, there is the application of an anaerobic-feast phase followed by an aerobic-famine phase [17,18]. This promotes the growth of storage microorganisms such as Phosphorus Accumulating Organisms (PAOs) and Glycogen Accumulating Organisms (GAOs) [14]. Research has revealed that selecting for microorganisms with a low maximal growth rate, such as PAOs and GAOs, would lead to improved granule stability [19,20]. Enhancement of this effect could be achieved by feeding the substrate under anaerobic conditions, allowing only the storage of substrate without growth. It has been shown that this indeed leads to stable granule formation at low oxygen concentrations, with the capability to simultaneously remove carbon, nitrogen, and phosphorus. For this purpose, the proper stratified structure of the granular sludge offers optimal redox conditions for the removal of organic matter, phosphorus, and nitrogen by performing, for the latter, a simultaneous nitrification denitrification (SND) [12].

Bearing in mind the above, the combination of both MBR and AGS technologies is the origination of the AGS–MBR process, which combines advantages of both the technologies in terms of removal efficiencies, and could offer some advantages in fouling mitigation if the granules directly collide with membrane fibers [10,11,21,22,23,24,25,26,27,28,29,30].

This mini-review is aimed at providing an updated critical literature review about the AGS–MBR configuration process. Particular attention is paid to: (i) different process configurations, such as separated or single reactors; (ii) the effects of AGS towards membrane-fouling, focusing on the incidence of granule mean dimensions and extracellular polymeric substances (EPS) of granular sludge on fouling deposition; and (iii) future perspectives of AGS–MBR technology.

## 2. Process Configurations

**Figure 2:**
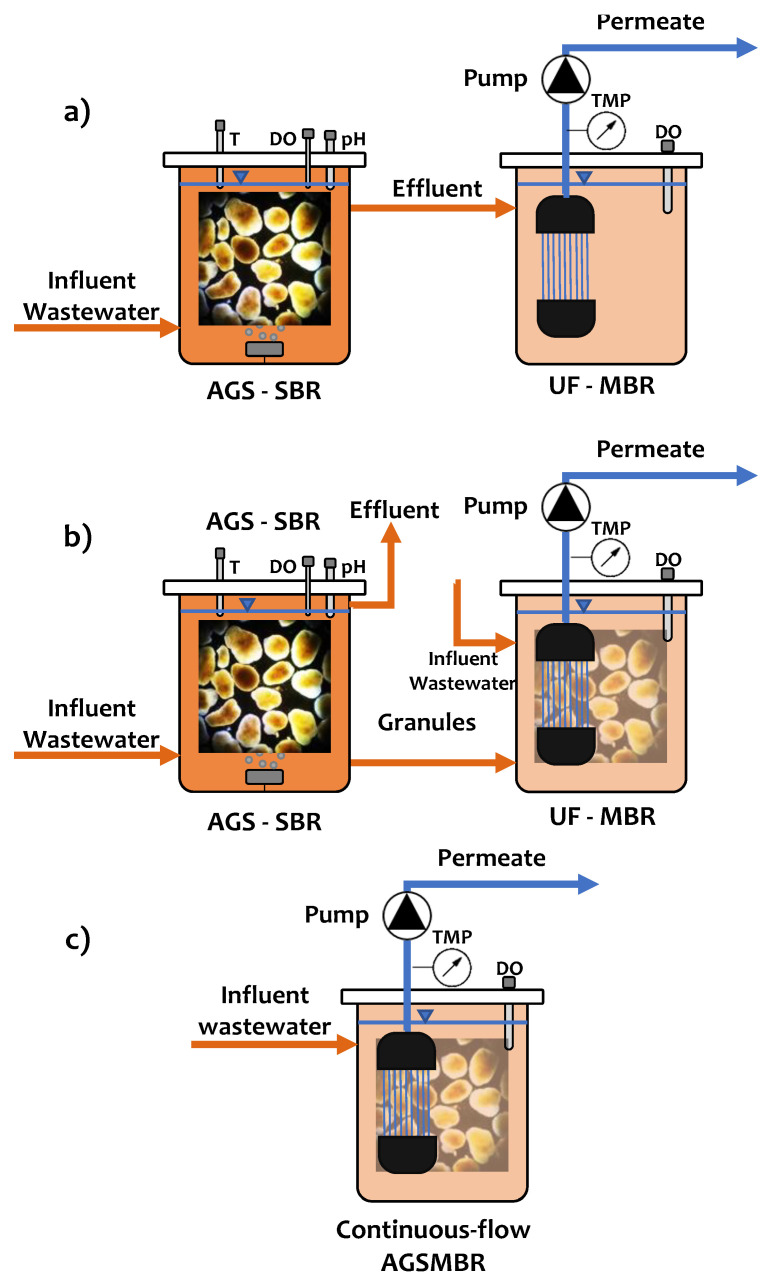
Process configurations of AGS–MBR systems: (**a**) Submerged MBR fed with the effluent of a separated granular SBR; (**b**) Granular sludge cultivated in a separated SBR, fed to submerged MBR; (**c**) continuous-flow AGS–MBR with granules in direct contact with membrane fibers.

As mentioned in the previous paragraph, a condition of primary importance for the formation of stable aerobic granular sludge is represented by the alternation of the substrate feast/famine phases, commonly obtainable from a sequential operation (SBR) of the reactor [19]. For this reason, some AGS–MBR process configurations follow the scheme with separate reactors SBR–MBR (Figure 2a), where, in the first SBR reactor, there is the formation of stable granules and the bulk of the process, while in the second submerged reactor MBR is fed with the effluent of the SBR reactor and will act as tertiary treatment for solid–liquid separation of the suspended solids [23,31,32,33,34]. Another widespread plant configuration always provides for the presence of an SBR reactor for the cultivation/maintenance of aerobic granules, followed by a submerged MBR reactor fed with mixed liquor containing aerobic granules. In this case, the aerobic granules come into direct contact with the membrane fibers (Figure 2b) [22,35,36,37,38]. The most applied configuration for AGS process on a worldwide scale is in sequencing batch reactors (SBR) [12], where HRT strictly depends on volumetric exchange ratio (VER) and MBRs work in continuous flow. In this case, the most applicable configuration for the AGS–MBR process could be that shown in Figure 2b, where the two technologies can only work in separated reactors. To work in a single reactor, a viable way could be to work AGS in continuous-flow mode. In this case, the AGS–MBR process operates in a continuous flow and the aerobic granules come into direct contact with the membrane fibers (Figure 2c) [10,21,24,25,27,28,39,40,41,42,43,44,45,46,47,48,49,50,51,52]. To date, the continuous-flow operation of AGS reactors is still a much-debated topic in the literature, and the main issue is represented by maintaining the stability conditions of granules in a configuration that does not normally include the alternation of the feast/famine phases [45]. To overcome this drawback, it is possible to apply proper hydrodynamic measures to obtain the feast/famine regime in space and not in time, as for SBR [45,53].

Table 1 reports an updated literature review of the aforementioned process schemes of AGS–MBR, including the main operational parameters and the features of each research.

## 3. Removal Efficiencies of AGS–MBR

Regarding the removal efficiencies of the separated reactors (SBR–MBR) in AGS–MBR, Thanh et al. [31] obtained an organic matter removal efficiency higher than 96% in a wide range of OLR (2.5–15 kgCOD/(m^3^d)). A few years later, the same authors in another experiment [23] with a similar reactor configuration obtained a 97.3% dissolved organic matter (DOC) removal efficiency and a 59% total nitrogen (TN) removal efficiency. The latter was achieved through simultaneous nitrification denitrification (SND) in the granular SBR, due to granule redox oxidation-reduction potential (ORP) stratification. Vijayalayan et al. [34] obtained 99% of total organic carbon (TOC) removal efficiency, and 61% TN removal efficiency. The latter was mainly limited by e-donor availability. Simultaneous removal of organic matter and nutrients (TN and PO4-P) in a similar AGS–MBR configuration was reported by Iorhemen et al. [32]. The system achieved more than 98% COD removal, 96–99% TN removal, and more than 95% PO_4_-P removal. The remarkable removal of nutrients in this AGS–MBR configuration is attributed to the layered structure of aerobic granules, due to oxygen diffusion limitation as well as the proper metabolic selection of PAOs that stratified internally in the granule’s layers. When treating industrial wastewater (i.e., citrus wastewater), Di Trapani et al. [33] obtained high organic matter removal efficiency close to 95% as COD, similar to the efficiency obtained with traditional MBR.

Concerning the removal efficiencies of the separated configuration with AGS and submerged MBR, Zhou et al. [35] reported a 95% COD removal efficiency, and a 90% TN removal efficiency via SND achieved in the granular SBR. High COD removal efficiency (99%) was also obtained by Tay et al. [54] for both AGS–MBR and traditional MBR. In a sequencing of AGS–MBR, Tu et al. [37] achieved high removal efficiencies in terms of organic matter removal (up to 98%) and nitrogen removal (83–86%).

With reference to the single-reactor AGS–MBR configuration, Li et al. [49] and Li et al. [51] obtained about 80–95% and 85–90% of COD removal efficiency, respectively. Wang et al. [39] reported a TOC removal efficiency in the range 84.7–91.9% and a total nitrogen removal efficiency in the range 41.7–78.4% for a continuous-fed AGS–MBR. Yu et al. [28] registered a COD removal efficiency >85%, while Li et al. [25] and Juang et al. [26] reported a COD removal efficiency >95% and of 91%, respectively. The latter also registered a nitrification efficiency of 96%. Liu et al. [24] reported 83%, 67% and 60% for COD, phosphorous, and nitrogen, respectively. High removal efficiencies were also observed by Zhang et al. [38], who reported a COD removal efficiency higher than 98% for two AGS-MBRs equipped with different membrane material (i.e., PVDF and PTFE). A nitrification efficiency close to 99% and an TN removal higher than 66% were registered for both the configurations.

Excellent organic matter removal efficiency (i.e., close to 90% for COD) was observed in a continuous-flow AGS–MBR [45]. Scarce nutrient (i.e., nitrogen and phosphorus) removal efficiencies were registered after granule breakage, due to the loss of the granule layer. Chen et al. [46] obtained high removal efficiency (i.e., >80%) for both organic matter and nitrogen. Recent studies [11,48] revealed that in a continuous-fed AGS–MBR, it is possible to achieve high organic matter removal efficiency close to 96%, medium nitrogen removal efficiency close to 50%, and medium–low phosphorus removal efficiency close to 35%.

## 4. Fouling Behavior and Analysis in AGS–MBR Systems: Better or Worse Than Traditional MBR?

As reported in the introduction, fouling is the most important drawback for MBR systems, restraining the filtration efficiency and enhancing operational cost [3]. Fouling is commonly monitored through daily trans-membrane pressure (TMP) registration, the evaluation of the fouling rate (FR) expressed as increase of TMP of the total resistance over time. Furthermore, some studies report a more refined fouling interpretation taking into account the “resistance-in-series (RIS) model” [8,54]. According to this model, the total resistance to filtration (Rt or Rf) can be decomposed into multiple addenda, each referring to the specific fouling mechanism: cake deposition (Rc), which is often divided into reversible (Rc,rev), and irreversible (Rc,irr) resistances; and pore-blocking (Rpb), an irreversible resistance concerning the occlusion of the internal pores of membrane fibers.

**Figure 3:**
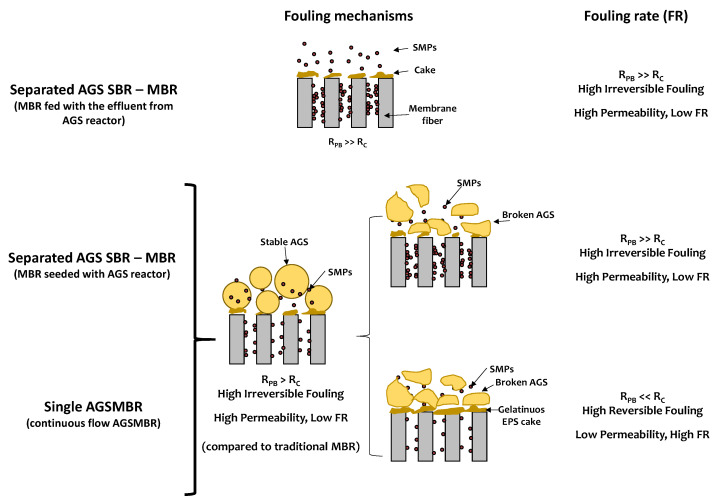
Fouling mechanisms in different AGS–MBR configurations.

In the following, an updated literature review of the AGS–MBR fouling formation and deposition is reported. In particular, each process configuration is analyzed, and Figure 3 shows a conceptual scheme of fouling mechanisms in AGS–MBR systems.

### 4.1. Separated Reactors (SBR–MBR)

Thanh et al. [31] analyzed the fouling behavior of the baffled membrane separation unit treating supernatant of a granulation SBR fed with synthetic wastewater. The OLR of SBR was 10–15 kgCOD/(m^3^·d) and the MBR unit was equipped with a flat-sheet polyethylene membrane with a pore size of 0.1 µm. Membrane-fouling of AGS–MBR was mainly characterized by pore-blocking, and Rpb was close to 59% of Rt. The principal cause of this irreversible fouling was represented by the soluble microbial products (SMPs) of the SBR supernatant, mainly composed of polysaccharides (84% of SMPs), identified as the main foulant that penetrates the membrane porosity. The same authors undertook similar research a few years later [23], with a similar layout: a submerged MBR compartment (polyethylene membrane with a pore size of 0.1 µm) was fed with the supernatant of a granular SBR to perform a treatment with continuity. It was confirmed that the polysaccharidic SMPs were the main causes of irreversible fouling due to pore-blocking. However, they stated that the AGS–MBR configuration showed a lower fouling rate compared to a traditional MBR (0.027 kPa/d), which permitted the extension of the continuous filtration duration (without backwashing) up to 78 days without any physical cleaning techniques. Vijayalayan et al. [34] worked with an MBR (polyethylene membrane with a pore size of 0.1 µm) fed with the effluent of a granular SBR. Also, in this work, the results confirmed that the dominant foulant was represented by polysaccharidic SMPs, which contributed to irreversible fouling. However, a granule breakage was registered due to the very high SRT of granules (300 days) that caused both the excessive growth of filamentous microorganisms and the lack of substrate and nutrient diffusion into the core of granules. This occurrence determined a sudden increase of FR from 0.105 kPa/d (before granule breakage), up to 0.475 kPa/d (after granule breakage). This experimental evidence confirmed the critical issue of AGS stability in AGS–MBR systems that could be preserved by operating within proper SRT values. In another study, Di Trapani et al. [33] studied a separated AGS–MBR process for the treatment of industrial wastewater (i.e., citrus wastewater). They observed that the effluent of the granular SBR was characterized by the presence of detached microorganisms and crushed granules, although the reasons for the AGS loss of stability were not addressed. This bulk composition resulted in a more hydrophobic cake layer that rapidly deposited on the membrane surface, thereby resulting in a rapid increase in the Rt in the short-term. Moreover, a similar cake layer was less compressible and more porous, resulting in a lower fouling rate. On the contrary, in such a condition, the mass transport of SMPs (proteins and polysaccharides) within the membrane internal pores will be favored. This circumstance promotes the increase of the irreversible Rpb in an AGS–MBR in separated configuration, as reported in previous works [23,31,34].

The configuration with a separated granular sludge SBR feeding an MBR leads to a low FR compared to a traditional MBR, but the fouling is more severe and is mainly irreversible, originated from pore-blocking.

### 4.2. Separated Reactors (SBR–Submerged MBR with AGS)

Regarding the submerged MBR fed with AGS from granular SBR, Zhou et al. [35] proved that the cake-layer resistance, Rc, in AGS–MBR was lower than the activated sludge MBR. This was likely due to the high back transport of granular sludge that enhanced the deposition of colloids and SMPs onto membrane fibers and into the pores. This led to severe pore-blocking (Rpb = 44%), compared to activated sludge MBR. In this case, the cake layer is more compact, less porous, and acts as a biological pre-filter able to biodegrade the organic foulants (i.e., EPS, SMPs) thus preserving membrane integrity. Interesting results were obtained by Tay et al. [36] that showed that AGS–MBR mixed liquor had better filtration properties than activated sludge MBR. Indeed, constant pressure tests indicated that when TMP increased 8-fold, the membrane permeability loss in AGS–MBR was 1.68-fold lower than traditional MBR. Constant flux test showed that when flux increased 3-fold, the loss of membrane permeability in AGS–MBR was 21-fold lower than traditional MBR. Furthermore, during continuous reactor operation, membrane TMP in traditional MBR increased periodically to 50–60 kPa and regular physical cleaning was required. In AGS–MBR, TMPs were at least one order of magnitude (3–6 kPa) lower than traditional MBR and no physical cleaning was required. Moreover, the study remarked that the contribution to fouling due to soluble products was similar in AGS–MBR and MBR. The much better filtration characteristics of AGS–MBR mixed liquor was due to the lower compressibility of its biomass, dominated by aerobic granular sludge. However, the filtration characteristics of the AGS–MBR system can suddenly worsen if the AGS stability is not preserved. Tu et al. [37] tested a submerged sequencing AGS–MBR. In this work, the AGS was cultivated inside the sequencing AGS–MBR, obtaining a complete granulation after 270 days of operation (granule dimeter > 300 µm). The increase of membrane-fouling after granulation was low, with a fouling rate close to 0.03 kPa/d (TMP below 8 kPa). The increase of the PN/PS ratio of mixed liquor after granulation caused an increase of hydrophobicity and an improvement of the filterability. Membrane-fouling is closely linked to sludge morphology/structure and AGS was beneficial for slowing down the fouling rate and prolonging the membrane permeability, as stated by Wang et al. [22], who reported an almost steady increase of TMP for AGS–MBR compared to the exponential-like increase in the case of flocculent MBR. In general, pore-blocking was found to be the dominant mechanism in membrane-fouling for AGS–MBR, highlighting that Rpb was the key factor and contributed to about 76% of total resistance to filtration. Aerobic granules have a larger size than the pores of the membrane and do not attach to the membrane surface easily. The formed cake layer is characterized by a loose structure that would benefit the overall operation of AGS–MBR resulting in a low fouling rate. However, despite the slow fouling rate, pore-blocking was the main factor for membrane-fouling. This tendency was the opposite compared to a traditional activated sludge MBR, where the compactness of the flocculent cake layer led to a prevalence of cake-fouling (Rc ≈ 61% of Rt). Moreover, with EPS account as the main foulant, its composition in terms of PN/PS was decisive. Indeed, AGS was more hydrophobic than flocculent sludge, which was more hydrophilic. This evidence was confirmed by the different PN/PS ratio of the EPS of both the sludges (PN/PS > 1 for AGS, PN/PS < 1 for flocculent sludge), considering that PN were hydrophobic whereas PS were hydrophilic. Therefore, AGS was intrinsically more hydrophobic than flocculent sludge, and this resulted in a less compact and less compressible cake layer in an AGS–MBR configuration. These results were confirmed by Zhang et al. [38], who reported that the cake layer formed by the AGS was porous, compared to a cake from flocculent activated sludge. This could not prevent small foulants from entering the membrane pores, leading to the blocking of the membrane pores. Furthermore, comparing the PTFE and PVDF membranes, the contents of the EPS and microbial communities on the membranes differed widely depending on different membrane material, and the higher PN contents of the EPS and SMP on the PVDF membrane resulted in more serious fouling. Rpb/Rf ratios of the PVDF and PTFE membranes were 59.8% and 56.4%, respectively, which were higher than the corresponding Rc/Rf values. Therefore, the PTFE membrane had better antifouling performance than the PVDF membrane. Furthermore, after physical and chemical cleaning, the PTFE membrane exhibited a higher flux-recovery rate than the PVDF membrane, indicating its superior antifouling performance.

### 4.3. Single Reactor (AGS–MBR)

The first study taking into account the application of AGS–MBR technology in a continuous-flow feeding mode was conducted by Li et al. [49]. Also, in this configuration, it was reported that the main fouling mechanism was the pore-blocking, due to the lack of a proper cake layer acting as a pre-filter, although the permeability loss was lower than a traditional flocculent MBR. Therefore, the pore-blocking accounts for the main fouling resistance in AGS-membrane filtration. Later, the same research group concluded that the introduction of aerobic granular sludge into MBR could alleviate membrane-fouling and the membrane permeability was 50% higher than that of a membrane bioreactor with floc sludge, despite the increase of internal membrane pore occlusions [50]. One year later, similar results concerning the dominance of pore-blocking fouling in a AGS–MBR were obtained by Juang et al. [26,40]. Li et al. [51] focused on the biogranulation in a continuous-fed AGS–MBR and the relationship with granules stability and membrane-fouling. As reported in the previous sections of the present work, one of the principal factors for a successful and stable granulation is represented by the alternation of feast/famine regime that enhance EPS production and consumption during the starvation. In a continuous-fed AGS–MBR configuration, the mechanisms for EPS production are different and while in a GSBR EPS are cyclically produced and consumed in the feast/famine regime, in a continuously fed AGS–MBR the continuous cut-off of EPS by membranes and the lack of starvation made the EPS concentration increase in sludge and in the supernatant. However, EPSs are known as one of the worst foulants for MBR [3], therefore a proper process management (for instance by regulating the SRT of aerobic granules) should be performed. Wang et al. [39] confirmed that the drastic change of operating mode from SBR to continuous MBR (i.e., the lack of feast/famine regime) can be harmful/fatal for granule stability. Indeed, a partial disaggregation of granular sludge inoculated into a continuous-flow AGS–MBR was observed. This drawback can be determinant in the increase of membrane-fouling and in the loss of nitrogen and suspended solid removal efficiencies. Yu et al. [28] reported a low TMP (<70 kPa) and they attributed this to the lower sludge resistance to filtration (SRF) for granular sludge (1.5–4.9 × 10^11^ m/kg), compared to flocculent sludge (4.6 × 10^12^ m/kg). In another work, Li et al. [25] worked with a mesh filter MBR (nylon and porosity of 70 µm) and highlighted that despite the concept to which EPS are strictly correlated with fouling resistance of MBR [3], in an AGS–MBR the cake porosity or structure can be more important than cake composition. Therefore, the AGS–MBR registered a very low TMP (close to 0.25 kPa) denoting an almost reversible fouling also due to the high porosity of mesh filter (70 µm). An experience with the combination of AGS technology and dynamic MBR (pore size 100 µm) was conducted by Liu et al. [24]. After a hard continuous operation of the dynamic membrane for more than a month, the membrane resistance had no obvious increase, thus demonstrating that membrane-fouling could greatly be reduced by introducing granular sludge in the dynamic MBR. Sajjad et al. [43] studied the hydraulic performance of a continuous-flow membrane bioreactor (CFMBR) where AGS was cultivated. An increase of TMP up to 90 kPa was found after 90 days of continuous filtration. Low fouling rate of 0.25 kPa/d, without any membrane cleaning, was registered. The high PN/PS ratio (3.3) in the GSBR compared to the CFMBR enabled the granular sludge to increase its filtration rates due to the hydrophobic nature of the proteins. However, the relatively higher number of hydrophilic polysaccharides in CFMBR lowered the PN/PS ratio, which subsequently decreased the sludge dewaterability. The granular sludge filterability in AGS–MBR was nearly three times higher than the flocculant sludge of this reactor. The granule formation in this continuous-flow system lessened the concentration of sludge flocs, which resulted in the alleviation of membrane-fouling. The periodic renewal of granules significantly delayed the frequency of membrane cleaning. However, the study did not report the typology of fouling (reversible or irreversible fouling), nor observed any loss of stability in aerobic granules. Iorhemen et al. [9,55] remarked that the major technical problem of AGS–MBR systems is the long-term system operation instability of aerobic granulation and granule disintegration problems. Indeed, the breakage of granules impacts the efficiency of wastewater treatment in the long-term operation, and is a critical issue in full-scale operations. Granule disintegration increases the concentration of soluble EPS, consequently increasing the irreversible membrane-fouling (i.e., pore-blocking). Corsino et al. [45] proposed a continuous-flow reactor with a novel geometric configuration, aiming at clarifying the mechanisms linked to the stability of AGS in a continuous-fed AGS–MBR in terms of structural characteristics and biological performance. A particular layout was designed to achieve the feast/famine regime physically in the space in the continuous-flow reactor, given that in an SBR the feast/famine regime occurs along the SBR cycle time. This work opened a new possible scenario in AGS–MBR technology, since it was reported that the pore-blocking resistance (Rpb) was about one order of magnitude lower than the irreversible cake that was removable with proper physical cleaning [8], so no chemical cleaning was necessary. This was an important novelty, compared to previous studies of AGS–MBR [9], where pore-blocking fouling was dominant. However, in this experiment, a huge loss of granule stability was observed. The granule breakage determined an irreversible fouling resistance, mainly represented by a compact and hydrophobic cake, composed of gelatinous EPS form broken AGS. A smaller fraction of SMP produced by substrate use was responsible for a residual pore-blocking of membrane fibers. The principal reason for the failure of granule stability is attributable to high granule SRT (i.e., about 50 days) in a continuous-flow AGS–MBR [45]. Indeed, by operating at high SRT, the growth of filamentous microorganisms inside the granule structure is possible [56]. This occurrence could cause the breakage of AGS, and operating at lower SRT could enhance the maintenance of AGS structure [45]. Chen et al. [46], did not observe severe fouling in their continuous-flow AGS–MBR with granules cultivated inside the system. Iorhemen et al. [11,48] observed that in a continuous-fed AGS–MBR it is possible to obtain a gentle TMP rise due to the sloughing of the cake layer through the abrasion by AGS. Moreover, they observed that the rise in TMP (up to 46 kPa) is due to the high protein content in soluble EPS. However, TMP was low, despite the registered high PN/PS ratio. Zhang et al. [47] discussed a novel issue for membrane-fouling in AGS–MBR systems linked to the granule sizes, finding that there is a critical size (1~1.2 mm mean diameter) with the highest membrane-fouling. Below that, the cake-fouling layer is tight and high compressibility emerged, while some pore-blocking occurs. Above that, this cake-fouling layer becomes loose and highly permeable, and more EPSs emerge. Working in this critical size, membrane-fouling is the most serious, because of the dual role of the compact structure of cake-fouling layer and the adhesion of EPS. The “antifouling” ability of AGS can be effectively maximized by avoiding or keeping away from the critical size. In a recent work, Song et al. [21] confirmed that granular sludge exhibited significantly lower fouling (i.e., lower fouling rate) potential than conventional activated sludge in AGS–MBR also under high salinity environment. The bigger size of granular sludge induced higher shear transport, which overwhelmed the filtration dragging force and foulant–membrane interaction, consequently leading to less deposition on the membrane surface. The most recent work dealing with AGS–MBR was conducted by Zhang et al. [10], focusing on the effect of scouring on fouling mitigation. A new hydrodynamic model was developed to explain the scouring mechanism of AGS. The scouring stress, proportional to the total amount of AGS depositing on the membrane surface, effectively reinforced the collision between AGS and membrane and reduced their deposition on the membrane surface by friction with the membrane. Thus, it was further conducive to membrane-fouling mitigation. Moreover, a novel contribution quantification model was proposed for analyzing the contribution rate of AGS scouring effect to mitigate membrane-fouling. AGS scouring possessed a significant contribution rate (39.9%) for fouling mitigation, compared with AGS structure (50.3%) and hydraulic stress (9.7%). In conclusion, this study provides an in-depth understanding to mitigate MBR membrane-fouling by the unique advantages of sludge granulation.

In summary, when the AGS encounters the membrane unit and granules preserve their structural stability, both in separated SBR–MBR reactors and in a single AGS–MBR reactor, the cake layer is more porous and swollen, leading to a higher permeability (low FR) and a higher irreversible fouling (i.e., pore-blocking) compared to the traditional MBR (Figure 3). If AGS loses its structural stability, the two main cases of fouling behavior can be identified: (i) a severe irreversible fouling due to pore-blocking, but a low FR; and (ii) a high reversible fouling, due to cake-layer deposition of gelatinous EPS of broken AGS, which leads to a high FR.

## 5. Future and Perspective of AGS–MBR Technology

AGS–MBR technology offers great potential for fouling mitigation and wastewater treatment in traditional MBR processes [9]. The main advantage was provided by AGS towards membrane cake deposition, thus acting as a “fouling retardant”. Indeed, the continuous rubbing of aerobic granules with membrane fibers involved a sensible lowering of fouling rate as TMP increases versus time [10]. Many studies [9,43,47] refer to a contextual worsening of irreversible fouling (i.e., pore-blocking) due to the high presence of SMPs in the bulk that penetrates inside the porosity of membranes, both for the separated configuration (two reactors SBR–MBR) and the combined configuration (single-reactor AGS–MBR). This was due to the higher SMP production in an AGS system than an activated sludge system, and to the occurrence of granule instability problems that led to the disintegration of granules in the long term [9]. However, from the literature review [10,45,46,47], it is possible to point out four main possibilities to develop stable AGS–MBR systems in the future, preventing the growth of irreversible pore-blocking in the membrane filtration unit: (i) cultivate AGS directly in the new AGS–MBR configuration; (ii) ensure the occurrence of anaerobic-feast/aerobic-famine regime in the continuous-flow reactors; (iii) control granule mean dimensions such that they are far from critical values (1~1.2 mm); and (iv) manage AGS scouring.

Cultivating AGS directly in the new AGS–MBR system has the advantage of physically and metabolically selecting the granules according to the new operational conditions [46], thus obtaining stable and durable aggregates. When AGS is seeded to the AGS–MBR from a separated cultivation SBR, the granules underwent a drastic variation of environmental and operational conditions that hampered the maintenance of structural stability during time. If AGS is formed inside the AGS–MBR configuration, the durability of granules is more probable. Furthermore, as almost all the single-reactor AGS–MBR configurations are fed in continuous-flow mode, a crucial operational condition to promote/preserve granule stability is the occurrence of anaerobic-feast/aerobic-famine regime [45]. This circumstance is fundamental both in the cultivation phase of granules, to select slow-growing organisms (i.e., PAOs and GAOs) that will enhance the formation of AGS, and in the operation phase to outcompete the fast-growing flocculent organisms (i.e., ordinary heterothrophic organisms, OHOs) thus ensuring the maintenance of granule structural stability. Proper management of granule size, far from the critical mean diameter of 1~1.2 mm in an AGS–MBR, is considered an important factor to mitigate membrane-fouling [47]. In particular, the irreversible fouling caused by pore-blocking is inversely proportional to granules sizes. Therefore, to minimize pore-blocking, having AGS sizes above the critical range is beneficial. Furthermore, the overall stability of an AGS–MBR system can be guaranteed by keeping a proper scouring effect of AGS towards membrane fibers [10], thus ensuring a fouling control and extending the durability of membrane. Finally, by comparing the AGS–MBR process to a traditional activated sludge MBR, it is possible to assert that the main advantage of AGS–MBR configuration is related to the possibility to operate with higher flux than an activated sludge MBR without observing a worsening of the permeability or fouling rate of the membrane. However, the management of an AGS–MBR process is more critical than traditional MBR due to the maintenance of structural stability of AGS and the mitigation of fouling through specific operational conditions (e.g., work far from the critical size of AGS; apply a proper scouring to membrane fibers), to avoid a severe irreversible fouling from pore-blocking in the long term.

## 6. Conclusions

The effect of AGS on performance and membrane-fouling in AGS–MBR systems was assessed.

MBR technology generates high-quality and largely disinfected effluent but membrane-fouling represents a major drawback hampering the wider application of MBRs due to the high costs associated with physical/chemical membrane cleaning. AGS is a novel kind of biofilm technology that offers several advantages, such as higher settleability, stronger microbial structure, higher resilience to toxic/recalcitrant compounds of industrial wastewater, high biomass concentration, and possibility to simultaneously degrade organic carbon and nutrients. For more than a decade, great interest has been expressed in the combination of AGS and MBR technologies resulting in AGS–MBR to mitigate membrane-fouling. However, granular sludge structural stability represents the main issue affecting AGS technology and granule breakage is a dramatic problem that implies an increase of irreversible (i.e., pore-blocking) membrane-fouling. From an updated literature review, four main factors were detected as the principal keys to develop stable AGS–MBR systems: (i) cultivate AGS directly in AGS–MBR configuration, instead of inoculating AGS from SBR; (ii) ensure the occurrence of anaerobic-feast/aerobic-famine regime in the continuous-flow reactors; (iii) verify that granules mean dimensions are far from critical values (1~1.2 mm); (iv) manage AGS scouring.

## Figures and Tables

**Table 1:** Literature review of AGS–MBR processes.

Process Configuration	Operation Mode	Wastewater	Granules Size (Average)	SRT	HRT	Biomass Concentration	Organic Matter Removal	P—Removal	N—Removal	TMP or Resistance to Filtration	PN/PS ratio of Bound EPS	Features	Ref.
			(µm)	(d)	(h)	(g/L)	%	%	%	kPa or m^−1^	-		
Submerged MBR with aerobic granular sludge (AGS) (PVDF—pore size 0.22 µm)	Continuous flow	Synthetic	590	n.d.	n.d.	n.d.	>90	>30	45	Fouling resistance to filtration (Rf) decreased from 5.70 × 10^12^ m^−1^, to 1.56 × 10^12^ m^−1^ due to the increase of AGS ratio that enhanced the cake permeability on account of AGS scouring effect, AGS structure and hydraulic shear.	n.d.	Membrane-fouling can be evidently mitigated after sludge granulation	[10]
Separated Sequencing Batch Reactor (SBR) and Submerged MBR (PVDF and PTFE pore size 0.1 µm)	Batch (SBR)—Continuous (MBR)	Synthetic	493 ± 36	25	12	6.7 as MLSS; 5.8 as MLVSS	>98	n.d.	>66	PTFE membranes had better antifouling performance, compared to PVDF membranes. Pore-blocking was the dominant form of membrane-fouling. R_pore_blocking_/R_fouling_ ratios of the PVDF and PTFE membranes were 59.8% and 56.4%, respectively, which were higher than the corresponding R_cake_/R_fouling_ values.	n.d.	The cake layer formed by the AGS was porous; it could not prevent small foulants from entering the membrane pores, leading to blocking of the membrane pores. PVDF membrane showed a higher PN contents of the EPS and SMP, compared with PTFE membrane, resulting in more serious fouling.	[38]
Submerged MBR with AGS inoculated with intertidal wetland sediment (IWS)	Continuous flow	Real saline pharmaceutical wastewater	3100–3300	10 (first 30 days); infinite (the last 90 days)	12	5 as MLSS	90	n.d.	31	Lower trans-membrane pressure (TMP) development rate, compared to conventional MBR.	n.d.	Granular sludge exhibited significantly lower fouling potential than conventional activated sludge in MBR under high salinity environment. The bigger size of granular sludge induced higher shearinduced transport, which overwhelmed the filtration dragging force and foulant–membrane interaction, consequently leading to less deposition on membrane surface.	[21]
Separated Sequencing Batch Airlift Reactor (SBAR) and Submerged MBR (PVDF pore size 0.04 µm)	Batch (SBAR)—Continuous (MBR)	Real industrial citrus wastewater	n.d.	1.8 (SBAR), 38 (MBR)	12 (SBAR), 53 (MBR)	6–8 as MLSS	95	n.d.	n.d.	Rapid increase of total resistance to filtration due to cake-layer deposition. Rapid increase of Fouling Rate (close to 10 × 10^12^ m/d)	n.d.	The AGS + MBR was characterized by higher values of total resistance to filtration and the fouling was characterized by a higher increase of irremovable fouling that can shorten the membrane life.	[31]
Submerged AGMBR—PVDF membranes (pore size 0.15 µm)	Continuous flow	Synthetic	n.d.	25	6,8,10	7.9 ± 1.7 as MLSS	96	35	50	Gentle TMP rise due to the sloughing of the cake layer through the abrasion by AGS.	2–16	The rise in TMP (up to 46 kPa) is due to the high PN content in soluble EPS. TMP rise was low despite the high PN/PS ratio	[11,45]
AGS reactor—Side-stream PVDF membrane (pore size 0.15 µm)	Continuous flow	Synthetic	n.d.	n.d	n.d	4.3 as MLSS	n.d.	n.d.	n.d.	n.d.	n.d.	Critical AGS size (1–1.2 mm) for membrane-fouling. Exceeding 1.2 mm, flux rose and fouling decreased with size, since the loose cake layer formed by larger AGS demonstrated a high permeability. Less than 1 mm, better flux and smaller fouling emerged at lower size, due to less EPS production. As for the critical size, the highest fouling was caused by the dual role of the compact structure of cake-fouling layer and the adhesion of EPS.	[44]
AGS reactor—Side-stream PVDF membrane (pore size 0.10 µm)	AGS SBR—continuos flow MBR	Synthetic	n.d.	n.d	n.d	9.2 as MLSS	98	≥95	96–99	n.d.	n.d.	n.d	[30]
Submerged aerobic granular sludge MBR—PVDF membrane (pore size 0.22 µm)	Continuous flow	Synthetic	n.d.	110	5	6–8 g/L	>80	n.d.	>80	membrane cleaning when TMP reached 30 kPa	always <1– > dominance of PS content	n.d	[43]
*continue*													
Submerged aerobic granular sludge MBR—PVDF membrane (pore size 0.04 µm)	Continuous flow	Synthetic	n.d.	50	7.5	8	90	very low	very low	Rpb was an order of magnitude lower than the Rcake,irr due to low content of SMP in the bulk.	4–5	n.d	[42]
Submerged aerobic granular sludge MBR—PVDF membrane (pore size 0.22 µm)	Continuous flow	Synthetic with pharmaceuticals	n.d.	20	4	5.1 as MLVSS	92	90	88	n.d.	n.d.	The removal rates of prednisolone, norfloxacin and naproxen reached 98.5, 87.8 and 84%, respectively. The degradation effect in the GMBR system wasrelatively lower for sulphamethoxazole and ibuprofen, withremoval efficiency rates of 79.8 and 63.3%, respectively.	[41]
Continuous-flow membrane bioreactor (CFMBR) seeded with aerobic granular sludge (AGS)	Continuous flow	Real wastewater	550	n.d.	8	3.5 as MLSS	n.d.	n.d.	n.d.	TMP = 20 kPa after 90 days of continuous filtration. Low fouling rate of 0.25 kPa/d, without any membrane cleaning.	3.3	The granular sludge filterability in CFMBR wasnearly three times higher than the flocculant sludge of this reactor. Thegranule formation in CFMBR lessened the concentration of sludge flocs, which resulted in the alleviation of membrane-fouling. The periodic renewal of granulessignificantly delayed the frequency of membrane cleaning.	[40]
Submerged aerobic granular sludge MBR -Polyethylene membrane (pore size 0.01 µm)	Continuous flow	Synthetic with pharmaceuticals		30	2	n.d	92.7	90 (as NH4-N)	n.d.	n.d.	n.d.	The removal rates ofprednisolone, naproxen, and norfloxacin were 98.56, 84.02,and 87.85%, respectively. The removal rates of sulfamethoxazoleand ibuprofen were 77.83 and 63.32%, respectively	[39]
Submerged aerobic granular sludge MBR—PVDF membrane (pore size 0.02 µm)	Continuous flow	Synthetic with pharmaceuticals		n.d.	n.d.	4.1 as MLVSS	80–90	90	95	n.d.	n.d.	Removal rate of prednisolone (98%), naproxene (84%), ibuprofene (63%), amoxicillin (irrelevant).	[38]
Batch Granulation Membrane Aerated Bioreactor (BG-MABR)—Separated Sequencing Batch Airlift Reactor (SBAR) and Membrane Airlift Bioreactor MABR Polyethylene (pore size 0.1 µm)	Batch	Synthetic	1700	24 (SBAR), 40 (MBR)		7.6 aa MLVSS (SBAR); 3.9 as MLVSS (MABR)	99	n.d.	61	Low fouling rate of 0.105 kPa/day	0.17	The deflocculation and lysis processes are the main sources for generation of soluble EPS in the system. The advantages of the granular sludge as well as the MABR sludge in terms of good settling when compared to the conventional MBR favors the use of MABR coupling with the granulation reactor. Approximately, 30% and 50% of the soluble PS and PN were retained by the membrane which shows that the remaining PS and PN were deposited on pores and surface of the membrane. This phenomenon has caused irreversible fouling in the membrane.	[32]
Submerged aerobic granular sludge MBR—PVDF membrane (pore size 0.4 µm)	SBR	Synthetic	1000	8	n.d.	3–10 as MLSS	n.d.	n.d.	n.d.	Good and stable aerobic granules greatly retarded the membrane-fouling, thus contributing to a gentle TMP rise. The pore-blocking resistance (Rpb) close to 76.21% was the key fouling factor for aerobic granular sludge MBR.	2.5	The pore-blocking resistance was the main factor inaerobic granular sludge. The AGMBR allowed 61 days of filtration without the need for cleaning, a higher value if compared with 10, 14, and 19 days for bulking, flocculent, and small granular sludge, respectively. Granules were stable during operation.	[22]
Batch Granulation Membrane Bioreactor (BG-MBR)—Separated Sequencing Batch Airlift Reactor (SBAR) and Submerged MBR Polyethylene (pore size 0.1 µm)	Batch (SBAR)—Continuous (MBR)	Synthetic	4900	24 (SBAR), 20 (MBR)	7.3 (SBAR), 3.4 (MBR)	12.6 as MLVSS (SBAR); 2.2 as MLVSS (MBR).	97.3	n.d.	59	Slow TMP rise, low fouling rate of 0.027 kPa/day.	1.7	Extended filtration period to 78 days without any need for physical cleaning. Granule were stable during the study period.	[23]
Submerged aerobic granular sludge MBR—microfiltration or ultrafiltration membrane widely used in MBR was substituted by a kind of silk with aperture of about 0.1 mm	Continuous flow	Synthetic		complete retention	13	10 as MLSS	83	67	60	After a hard continuous operation of the dynamic membrane for more than a month, the membrane resistance had no obvious increase, thus demonstrating that membrane-fouling could greatly be reduced by introducing granular sludge in the DMBR	n.d.	By combining the technologies of granular sludge and dynamic membrane, membrane-fouling could be greatly relieved.	[24]
*continue*													
Submerged aerobic granular sludge—mesh filter MBR nylon membrane (pore size 70 µm)	Continuous flow	Synthetic	500	32	6.7	5 as MLSS	91	96 (as NH4-N)	n.d.	Low TMP (0.24 kPa) during the stable operation period.	n.d.	Granules showed a lower fouling propensity than flocs, attributed to the formation of a biocake with more porosity than floc biocake.	[25]
Submerged aerobic granular sludge MBR (AGMBR)—Polyethylene membrane (pore size 0.4 µm)	Continuous flow	Synthetic	n.d.	n.d.	n.d.	n.d.	>95	n.d.	n.d.	Low TMP increase	n.d.	The AGMBR delays the occurrence of membrane-fouling when compared with the MBR tests; however, once fouling occurs, it was mostly contributed by irreversible fouling.	[26]
Submerged aerobic granular sludge MBR (GMBR)—PVDF membrane (pore size 0.22 µm)	Continuous flow	Synthetic	180–900	n.d.	n.d.	4 as MLSS	n.d.	n.d.	n.d.	TMP up to 17.8 kPa	Protein was the most predominantcomponent in EPS	Aerobic granules play an important role in reducing membrane pollutant	[27]
Submerged membrane sequencing batch reactor (MSBR) with aerobic granular sludge	SBR	Synthetic	500–1000	n.d.	n.d.	4–19 as MLSS	up to 98	83–86	n.d.	TMP below 8 kPa and fouling rate below 0.1 kPa/day	2–3	Membrane-fouling developed more slightly after sludge granulation was completed.	[35]
Aerobic granular sludge—Membrane bioreactor	Continuous flow	Synthetic	>5000	n.d.	24	TSS = 1.7 g/L; VSS = 1.5 g/L	>85	n.d.	n.d.	TMP below 70 kPa	n.d.	The quantities of proteins and polysaccharides in AGS increased first during granulation process, then declined owing to occurrence of intra-core anaerobic degradation.	[28]
Submerged aerobic granular sludge MBR reactor (GMBR)—PVDF membrane (pore size 0.22 µm)	Continuous flow	Synthetic	800–1500	35–45	5.3	4.2–5.9 g/L as MLSS	85–92	42–78	n.d.	n.d.	n.d.	Compared with SBR, the formation and stability of granular sludge are more complex in continuous GMBR than in SBR.	[36]
Submerged aerobic granular sludge MBR reactor (AGMBR)—Polyethylene membrane (pore size 0.4 µm)	Continuous flow	Synthetic	n.d.	n.d.	n.d.	n.d.	n.d.	n.d.	n.d.	n.d.	n.d.	The AGMBR exhibited a delayed TMP rise but, once occurred, irreversible fouling dominated the resistance.	[37]
Separated Sequencing Batch Airlift Reactor (SBAR) and Submerged MBR Polyethylene (pore size 0.1 µm)	Batch (SBAR)—Continuous (MBR)	Synthetic	300	n.d.	5.8 (SBAR), 12 (MBR)	n.d.	n.d.	n.d.	n.d.	n.d.	Soluble PS fraction (sPS), i.e., 84% of sEPS, as main contributor or membrane fouling.	Shell carrier was found to be an effective method in cultivating aerobic granule. Withstanding high OLR up to 15 kg COD/(m3 d).	[29]
Submerged aerobic granular sludge MBR reactor—PVDF membrane (pore size 0.2 µm)	Continuous flow	Synthetic	n.d.	n.d.	5	1.1–1.3 as MLSS	85–90	n.d.	n.d.	n.d.	0.6–1	The EPS released was closely associated with aerobic biogranulation in MBR system.	[48]
Submerged aerobic granular sludge MBR (AGMBR)—membrane pore size 0.1 µm	SBR	Synthetic	690–700	complete retention	8	6.5 as MLSS	99	n.d.	n.d.	TMP 3–6 kPa—No physical cleaning required.	n.d.	In AGMBR, membrane TMP of 3–6 kPa was maintained and no physical cleaning was required. The much better filtration characteristics of AGMBR mixed liquor was due to the low compressibility of its biomass, which was dominated by aerobic granular sludge. Membrane permeability loss (34.5%) in AGMBR was twice as low as the loss in the submerged MBR	[34]
Submerged aerobic granular sludge reactor MBR—Polyethylene membrane (pore size 0.1 µm)	Batch (SBAR)—Continuous (MBR)	Synthetic	500–1000	n.d.	n.d.	4.5 as MLSS		90	n.d.	R_pb_ is 44.2% of the membrane total resistance, which is higher than R_C_ proportion. Therefore, the membrane-fouling in the aerobic granular sludge was mainly due to the membrane pore-blocking during membrane filtration of granular sludge.	n.d.	The aerobic granular sludge could mitigate membrane-fouling significantly during short-term membrane filtration. However, the aerobic granular sludge could result in severe irreversible membrane-fouling.	[33]
*continue*													
Submerged aerobic granular sludge MBR (MGSBR)—Polypropylene membrane (pore size 0.1 µm)	Continuous flow	Synthetic	3000	60	5	15 as MLSS	n.d.	n.d.	n.d.	n.d.	n.d.	The introduction of aerobic granular sludge into MBR could alleviate membrane-fouling and the membrane permeability of MGSBR was more 50% higher than that of a membrane bioreactor with floc sludge.	[47]
Submerged aerobic granular sludge MBR (MGSBR)—Polypropylene membrane (pore size 0.1 µm)	Continuous flow	Synthetic	1000	complete retention	5	15 as MLSS	80–95%	n.d.	n.d.	TMP = 0.1 MPa	n.d.	During the period of operation, the membrane permeability of MGSBR was more 50% higher than that of a conventional MBR. The introduction of aerobic granules into the MBR system benefited for controlling membrane-fouling.	[46]

## Data Availability

Not applicable.
